# M7824, a novel bifunctional anti-PD-L1/TGFβ Trap fusion protein, promotes anti-tumor efficacy as monotherapy and in combination with vaccine

**DOI:** 10.1080/2162402X.2018.1426519

**Published:** 2018-02-14

**Authors:** Karin M. Knudson, Kristin C. Hicks, Xiaoling Luo, Jin-Qiu Chen, Jeffrey Schlom, Sofia R. Gameiro

**Affiliations:** a Laboratory of Tumor Immunology and Biology, Center for Cancer Research, National Cancer Institute, National Institutes of Health, Bethesda, MD, USA; b Collaborative Protein Technology Resource (CPTR), Center for Cancer Research, National Cancer Institute, National Institutes of Health, Bethesda, MD, USA

**Keywords:** PD-L1, TGFβ, carcinoma, checkpoint blockade, tumor microenvironment, TWIST1, vaccine, adenovirus

## Abstract

Tumors evade host immune surveillance through multiple mechanisms, including the generation of a tumor microenvironment that suppresses immune effector function. Secretion of TGFβ and upregulation of immune checkpoint programmed cell death ligand-1 (PD-L1) are two main contributors to immune evasion and tumor progression. Here, we examined the efficacy of a first-in-class bifunctional checkpoint inhibitor, the fusion protein M7824, comprising the extracellular domain of human TGFβRII (TGFβ Trap) linked to the C-terminus of human anti-PD-L1 heavy chain (αPD-L1). We demonstrate that M7824 reduces plasma TGFβ1, binds to PD-L1 in the tumor, and decreases TGFβ-induced signaling in the tumor microenvironment in mice. In murine breast and colon carcinoma models, M7824 decreased tumor burden and increased overall survival as compared to targeting TGFβ alone. M7824 treatment promoted CD8+ T cell and NK cell activation, and both of these immune populations were required for optimal M7824-mediated tumor control. M7824 was superior to TGFβ- or αPD-L1-targeted therapies when in combination with a therapeutic cancer vaccine. These findings demonstrate the value of using M7824 to simultaneously target TGFβ and PD-L1/PD-1 immunosuppressive pathways to promote anti-tumor responses and efficacy. The studies also support the potential clinical use of M7824 as a monotherapy or in combination with other immunotherapies, such as therapeutic cancer vaccines, including for patients who have progressed on αPD-L1/αPD-1 checkpoint blockade therapies.

## Introduction

In recent years, therapeutics targeting the immune system have become a major treatment modality in cancer. Monoclonal antibodies targeting immune checkpoints, such as programmed cell death-1 (PD-1) and programmed cell death ligand-1 (PD-L1), are a major class of these agents. The PD-1 receptor is expressed on activated T and natural killer (NK) cells. After interaction with its ligands PD-L1 and PD-L2, which are typically expressed on antigen presenting cells, PD-1 regulates immune responses by inhibiting T and NK cell maturation, proliferation, and effector function.^1^^,^^2^ Aberrant overexpression of PD-L1 has been observed in numerous malignancies and is associated with poor clinical outcome.^3^^,^^4^ Antibodies blocking PD-L1 or PD-1 inhibit this immunosuppressive pathway and have led to improvements in patient survival in melanoma,^5^^,^^6^ lung cancer,^7^^,^^8^ bladder cancer,^9^^,^^10^ and Merkel cell carcinoma,^11^^,^^12^ among others.^2^ Avelumab, which targets PD-L1, is approved for use in Merkel cell carcinoma and urothelial carcinoma. However, for most solid tumor types excluding melanoma, only 10–20% of patients typically respond to PD-1/PD-L1-targeted therapies.^2^ Therefore, improvement of these therapeutics is needed to increase patient response and survival.

In addition to expression of immune checkpoints, the tumor microenvironment (TME) contains other immunosuppressive molecules. Of particular interest is the cytokine TGFβ, which has multiple functions in cancer. TGFβ prevents proliferation and promotes differentiation and apoptosis of tumor cells early in tumor development. However, during tumor progression, tumor TGFβ insensitivity arises due to the loss of TGFβ receptor expression or mutation to downstream signaling elements. TGFβ then promotes tumor progression through its effects on angiogenesis, induction of epithelial-to-mesenchymal transition (EMT), and immune suppression.^13^^,^^14^ TGFβ mediates immune suppression by preventing the activation and division of T cells and decreasing effector function of both T and NK cells.^13^^,^^15^ In addition, TGFβ can induce the differentiation of regulatory CD4+ T cells (T_reg_),^16^^,^^17^ an indicator of poor prognosis for many tumor types.^18^^,^^19^ High TGFβ serum level and loss of TGFβ receptor (TGFβR) expression on tumors correlates with poor prognosis,^20-22^ which makes this pathway a noteworthy target for novel therapeutics.^23^^,^^24^ TGFβ-targeted therapies have demonstrated limited clinical activity. To our knowledge, however, none have targeted TGFβ inhibition or sequestration in the TME. TGFβ is a pleotropic cytokine involved in normal physiological function, which may be why small molecule inhibitors targeting TGFβ-dependent signaling have shown a high propensity for associated toxicities in preclinical models, including cardiac toxicities and the development of neoplasms.^23^

PD-L1 and TGFβ regulate immune suppression in the TME in distinct yet complementary ways. It is possible that targeting both the PD-L1 and TGFβ negative regulatory pathways simultaneously will increase anti-tumor efficacy. Here, we utilized a first-in-class bifunctional fusion protein designed to block PD-L1 and sequester TGFβ in the TME. M7824 (MSB0011395C) comprises the extracellular domain of human TGFβ receptor II (TGFβRII) linked to the C-terminus of the human αPD-L1 heavy chain, based on the human IgG1 monoclonal antibody (mAb) avelumab.^25^ To evaluate the PD-L1 versus TGFβ-targeted modes of action, we have also employed an M7824 mutant molecule devoid of the PD-L1 binding site. In this study, we evaluate the mechanisms of action and anti-tumor efficacy of M7824 in multiple murine tumor models and in combination with a therapeutic cancer vaccine.

## Results

### M7824 binds murine tumor PD-L1 in vitro and in vivo

**Figure 1.**
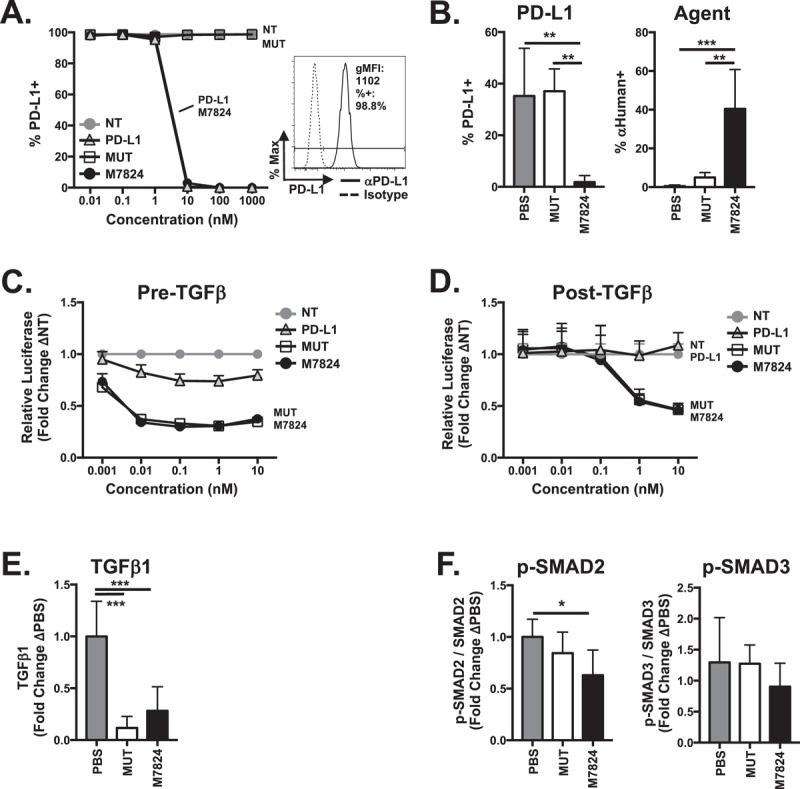
M7824 binds to murine PD-L1 and suppresses murine TGFβ signaling. (A) EMT6 tumor cells were treated with IFNγ for 24 hours to induce maximal PD-L1 expression (inset) followed by treatment with nothing (no treatment-NT), αPD-L1 (PD-L1), M7824mut (MUT), or M7824 for 30 minutes prior to analysis of surface PD-L1 expression by flow cytometry. Data represent 3 independent experiments. (B) 2.5 × 10^5^ EMT6 tumor cells were orthotopically implanted into Balb/c mice. When tumor volumes reached 50–100mm^3^, mice were treated i.p. at days 10, 12, and 14 with PBS or 492μg MUT or M7824. Twenty-four hours after the last treatment, intratumoral analysis of surface PD-L1 expression (left) and presence of biologic agents M7824mut or M7824 (right) on CD45 negative cells was performed by flow cytometry. Graphs show mean ± SD. Data represent 2 independent experiments, n = 5 mice. (C) 4T1-pSMAD2-luc tumor cells were exposed to PD-L1, MUT, or M7824 for 30 minutes followed by 2.5 ng/ml TGFβ1. Graph (mean ± SD) shows luciferase activity of SMAD2 reporter after 1 hour. Data represent 2 independent experiments. (D) 4T1-pSMAD2-luc tumor cells were treated with 2.5 ng/ml TGFβ1 for 30 minutes followed by PD-L1, MUT, or M7824. Graph (mean ± SD) shows luciferase activity of SMAD2 reporter after 6 hours. Data represent 3 independent experiments. (E) EMT6 tumor-bearing mice were treated as in (B). Twenty-four hours after the last treatment, plasma TGFβ1 level was examined. Graph shows mean ± SD. Data combined from 2 independent experiments, n = 3-6 mice per experiment. (F) EMT6 tumor cells were implanted as in (B). When tumor volumes reached 500mm^3^, mice were treated at days 17, 19, and 21 with MUT or M7824. Six hours after the last treatment, phosphorylation and total level of SMAD2 and SMAD3 were determined by capillary Western blot. Graphs show mean ± SD. Data combined from 2 independent experiments, n = 2-5 mice per experiment.

M7824 is a fully humanized molecule, so we first determined whether M7824 binds to murine PD-L1. A dose dependent loss of detectable surface PD-L1 expression was observed on EMT6 breast carcinoma cells after treatment with M7824, indicating its ability to specifically bind surface PD-L1 and with an affinity similar to that of avelumab, a fully humanized mAb targeting PD-L1 (hereafter referred to as αPD-L1) (Fig. 1A). The functional PD-L1 binding domain on M7824 was required for PD-L1 binding, as indicated by the use of a control comprising human TGFβRII bound to a mutated αPD-L1 moiety (referred to as M7824mut or MUT), which did not bind to PD-L1 (Fig. 1A). Similar results were seen using 4T1 breast and MC38 colon carcinoma cell lines (Supplementary Figure 1A). M7824 also bound to murine PD-L1 expressed in the TME on CD45-negative cells *in vivo* as indicated by the loss of surface PD-L1 staining. The functional anti-PD-L1 moiety was required for M7824 binding to TME-expressed PD-L1 as indicated by similar surface PD-L1 staining between M7824mut and PBS treatment (Fig. 1B, left panel). Direct detection of M7824 was performed using an anti-human antibody that specifically binds human IgG heavy and light chain. The functional αPD-L1 moiety was required for M7824 to accumulate in the TME, as M7824mut was detected at low levels in the tumor (Fig. 1B, right panel). Thus, M7824 is able to specifically bind PD-L1 both *in vitro* and *in vivo* and deliver TGFβ Trap to the site of the tumor.

### M7824 decreases tumor TGFβ signaling in vitro and in vivo and reduces plasma TGFβ1

There are three human and murine TGFβ isoforms, TGFβ1, TGFβ2, and TGFβ3. Binding of active TGFβ to the TGFβRI/TGFβRII receptor complex leads to phosphorylation and activation of canonical signaling molecules SMAD2 and SMAD3.^26^ To examine the ability of M7824 to sequester murine TGFβ and reduce TGFβ-dependent signaling, 4T1-pSMAD2-luc tumor cells, which express PD-L1 and TGFβRII and have intact TGFβ-dependent SMAD2/3 signaling (Supplementary Figure 1B, C), were used. Treatment of 4T1-pSMAD-luc tumor cells with M7824 either prior to (Fig. 1C) or after (Fig. 1D) the addition of TGFβ1 reduced TGFβ1-dependent phosphorylation of SMAD2, as indicated by decreased SMAD2 promoter-dependent luciferase activity (Fig. 1C, D). This effect was dependent on the TGFβ Trap portion of M7824, as M7824mut also reduced TGFβ-dependent signaling but αPD-L1 did not (Fig. 1C, D).

Treatment of EMT6 tumor-bearing mice with M7824 and M7824mut significantly reduced plasma levels of TGFβ1 after treatment (Fig. 1E), indicating their ability to bind to murine TGFβ1 *in vivo*. Plasma TGFβ2 and TGFβ3 levels were too low to detect in these studies. Importantly, M7824 also reduced intratumoral TGFβ signaling. Phosphorylation of SMAD2 in EMT6 tumors was significantly decreased 6 hours after M7824 treatment (Fig. 1F). Correlating with earlier results showing that M7824mut does not accumulate in the tumor (Fig. 1B), M7824mut did not reduce intratumoral SMAD2 signaling (Fig. 1F). M7824 treatment did not affect the phosphorylation of SMAD3 (Fig. 1F) or SMAD1 and SMAD5 (Supplementary Figure 1D) or activation of the non-canonical TGFβ-dependent signaling pathways ERK, JNK, or p38 (data not shown). These results support that M7824 sequesters murine TGFβ1 *in vitro* and *in vivo*. In addition, M7824 can both prevent the initiation of and significantly decrease existing TGFβ signaling, particularly in the TME.

### M7824 promotes activation of CD8+ T cells and NK cells in non-tumor-bearing mice

**Figure 2.**
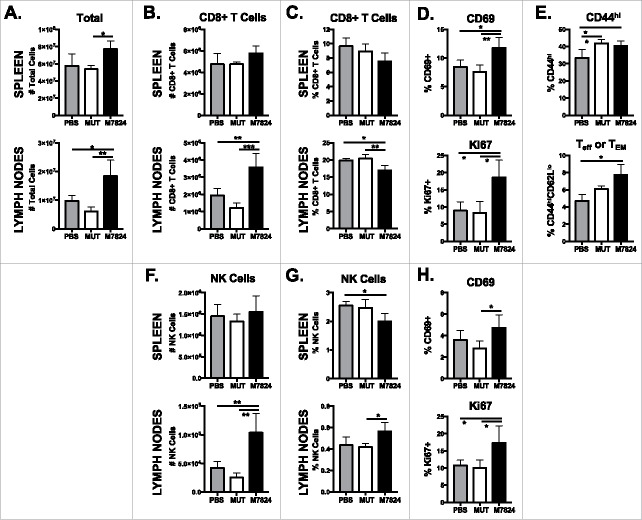
M7824 increases CD8+ and NK cell numbers in the lymph nodes and promotes their activation in non-tumor-bearing mice. Naïve Balb/c mice received MUT or M7824 i.p. on days 0, 2, and 4. Immune populations in the spleen and lymph nodes were analyzed by flow cytometry 3 or 7 days after the final treatment. Graphs show number of total cells (A), CD8+ T cells (B), and NK cells (F) or frequency (of total live cells) of CD8+ T cells (C), and NK cells (G) 3 days after last treatment. Phenotype of CD8+ T cells (D) and NK cells (H) was determined in the spleen 3 days after the last treatment. Maturation (CD44/CD62 L expression) of CD8+ T cells (E) was determined in the spleen 7 days after the last treatment. All graphs show mean ± SD. Data combined from 2 independent experiments, n = 3-5 mice per experiment.

TGFβ and PD-L1 affect T and NK cell phenotype and responses.^27^^,^^28^ In order to determine the direct effect of M7824 on immune cell populations, we first examined their phenotype in the spleen and lymph nodes of non-tumor-bearing mice after M7824 treatment. M7824 treatment increased total cell numbers in the spleen (Fig. 2A, upper panel) but did not affect splenic CD8+ T cell (Fig. 2B, upper panel) or NK cell (Fig. 2F, upper panel) numbers. In contrast, total cell (Fig. 2A, lower panel), CD8+ T cell (Fig. 2B, lower panel), and NK cell (Fig. 2F, lower panel) numbers increased significantly in the lymph nodes. Frequencies of CD8+ T cells (Fig. 2C) and NK cells (Fig. 2G) did not significantly increase in either the spleen or lymph nodes. A greater proportion of both CD8+ T cell (Fig. 2D, upper panel) and NK cell (Fig. 2H, upper panel) populations displayed an activated, CD69+ phenotype in the spleen. In addition, M7824 treatment increased the frequency of splenic CD8+ T cells (Fig. 2D, lower panel) and NK cells (Fig. 2H, lower panel) entering the cell cycle, as indicated by Ki67 expression. The frequency of splenic activated CD44^hi^ CD8+ T cells, specifically those with an effector or effector memory (T_eff_/T_EM_) CD44^hi^CD62L^lo^ phenotype, was enhanced upon M7824 treatment (Fig. 2E). Total CD4+ T cell numbers, but not frequencies, significantly increased in the lymph nodes of M7824-treated mice (Supplementary Figure 2A). CD4+ T_reg_ numbers also increased in both spleen and lymph nodes (Supplementary Figure 2B). In contrast to the changes seen with M7824, only minor differences were observed in all immune cell populations after treatment with M7824mut (Fig. 2, Supplementary Figure 2). These results indicate that M7824 promotes activation of CD8+ T and NK cells in non-tumor-bearing mice.

### M7824 has anti-tumor efficacy against breast and colon carcinomas

**Figure 3.**
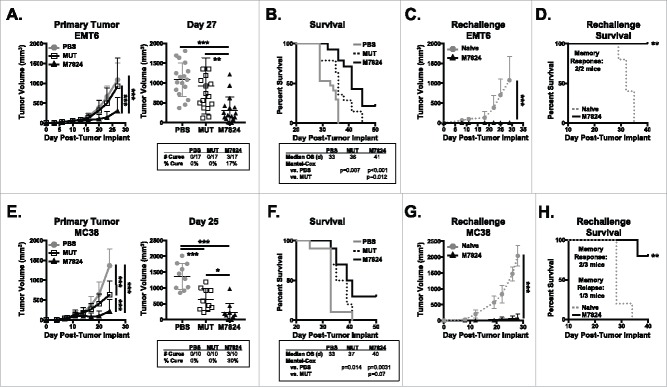
M7824 has anti-tumor efficacy against murine EMT6 breast and MC38 colon tumors. (A, B) EMT6 tumor cells were implanted as in Figure 1 and mice were treated at days 9, 11, and 13. Tumor volumes were measured and survival was tracked. (A) Primary tumor growth curves (left panel) and tumor volumes of individual animals (right panel, inset: number of cured mice) show mean ± SD. (B) Survival curves (inset: median overall survival (OS) in days) show % survival. (C, D) At least 1 month after tumor cure (day 94), cured mice and 5 naïve Balb/c were implanted with EMT6 tumor cells. (C) Tumor growth curves show mean ± SD. (D) Survival curves (inset shows # of mice with memory response) show % survival. Data are representative of 3 independent experiments, n = 10-20 mice. (E, F) 5 × 10^5^ MC38 tumor cells were implanted into female C57BL/6 mice and treated at days 9, 11, and 13. (E) Primary tumor growth curves (left panel) and tumor volumes of individual animals (right panel, inset: number of cured mice) show mean ± SD. (F) Survival curves (inset: median OS in days) show % survival. (G, H) At least 1 month after tumor cure (day 72), cured mice and 5 naïve C57BL/6 were implanted with MC38 tumor cells. (G) Tumor growth curves show mean ± SD. (H) Survival curves (inset: # of mice with memory response or relapse) show % survival. Data are representative of 2 independent experiments, n = 10 mice.

M7824 is able to effectively target both murine PD-L1 and TGFβ in the tumor microenvironment (Fig. 1B, F). We next investigated the therapeutic capacity of M7824 in multiple models of murine solid carcinomas. Administration of 20 mg/kg M7824, a clinically relevant dose,^29^ significantly reduced growth of EMT6 breast carcinoma tumors as compared to treatment with PBS or M7824mut (Fig. 3A). Seventeen percent of mice treated with M7824 underwent complete tumor rejection (Fig. 3A, right panel), which led to significant increases in median overall survival (OS) relative to mice treated with either PBS or M7824mut (Fig. 3B). No M7824mut-treated mice underwent complete tumor rejection (Fig. 3A). However, there was a trend of reduced tumor growth in M7824mut-treated mice with 29.4% of tumors being smaller than 500 mm^3^ at day 27 versus 11.7% in PBS-treated animals (Fig. 3A). This translated to a significant increase in OS relative to PBS treatment (Fig. 3B). EMT6 tumor rechallenge of M7824-cured mice did not produce palpable tumors (Fig. 3C, D), indicating M7824 promotes long-term memory protection against these tumors.

Similar results were seen in mice bearing murine MC38 colon carcinoma tumors. M7824 treatment significantly reduced tumor growth, cured 30% of tumor-bearing mice (Fig. 3E), and increased median OS versus PBS treatment (Fig. 3F). Unlike in the EMT6 breast carcinoma model, M7824mut also promoted a significant reduction in MC38 tumor growth compared to PBS treatment but to a lesser extent than M7824 (Fig. 3E). M7824mut treatment did not induce tumor rejection (Fig. 3E) or a survival advantage versus PBS (Fig. 3F). Rechallenge of M7824-cured mice showed a memory response in two of three mice (Fig. 3G, H). While protection from rechallenge was not as robust as against EMT6 tumors (Fig. 3C, D), these results suggest that M7824 does support long-term protective memory generation against MC38 tumors.

We also examined the effect of M7824 on spontaneous metastasis. Using the murine 4T1 triple negative breast carcinoma (TNBC) model, we observed that M7824 treatment was able to limit primary tumor growth relative to PBS-treated mice (Supplementary Figure 3A) and reduced 4T1 metastasis to the lung (Supplementary Figure 3B, C). Together, these studies show that dual targeting of TGFβ and PD-L1 with M7824 promotes significant anti-tumor efficacy in multiple murine breast and colon carcinoma models.

### CD8+ T and NK cells are responsible for the anti-tumor effects of M7824

**Figure 4.**
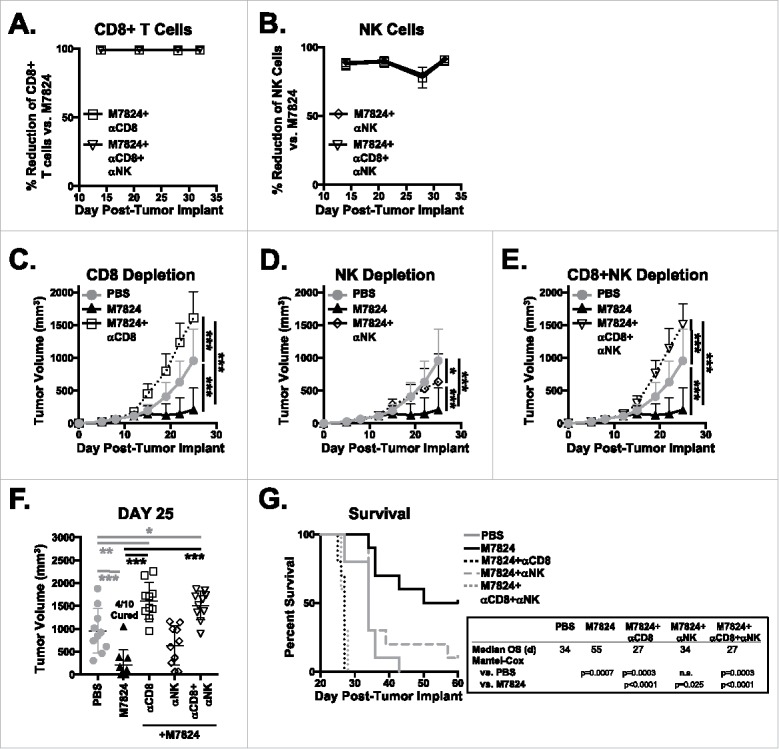
CD8+ T cells and NK cells are responsible for the anti-tumor efficacy of M7824. EMT6 tumor-bearing mice were treated as in Figure 1 with PBS or M7824. M7824-treated mice also underwent depletion of CD8, NK, or CD8 and NK cells. (A, B) Depletion efficiency was determined in the blood weekly by flow cytometry. Graphs show % reduction of CD8+ T cells (A) or NK cells (B) versus M7824-treated mice (set to 0%) as mean ± SD. (C, D, E) Primary tumor growth curves of mice that underwent CD8 (C), NK (D), or CD8 and NK (E) depletion show mean ± SD. (F) Graphs of tumor volumes of individual mice show mean ± SD. (G) Survival curves (inset: median OS in days) show % survival. Data represent 1 independent experiment, n = 10 mice.

Given that M7824 treatment activated both CD8+ T and NK cell populations in non-tumor-bearing mice (Fig. 2), we hypothesized that CD8+ T cells and NK cells promote the anti-tumor efficacy of M7824. To test this, we depleted CD8+ T cells and NK cells in EMT6 tumor-bearing mice treated with M7824. The depletion efficiency for CD8+ T cells (Fig. 4A) and NK cells (Fig. 4B) was approximately 99% and 75–90% during the course of the experiment, respectively. As seen in Fig. 3, treatment of EMT6 tumor-bearing mice with M7824 promoted significant reduction in tumor burden and increased median OS. Depletion of CD8+ T cells (Fig. 4C, F) and NK cells (Fig. 4D, F) completely abrogated the therapeutic capacity of M7824. NK cell depletion in M7824-treated mice led to tumor growth (Fig. 4D, F) and median OS (Fig. 4G) similar to that seen in PBS-treated animals. In contrast, depletion of CD8+ T cells significantly exacerbated tumor growth (Fig. 4C, F) and decreased survival by a week (Fig. 4G) as compared to PBS-treated mice. Tumor burden and survival of mice depleted of both CD8+ T cells and NK cells mimicked the results seen with CD8+ T cell depletion (Fig. 4E-G). These results suggest that CD8+ T cells are required for basal therapeutic efficacy of M7824 against EMT6 tumors and can partially compensate for the loss of NK cells but not vice versa. In all, these studies show that both CD8+ T cells and NK cells are required for the anti-tumor efficacy of M7824.

We also performed CD4 depletion on EMT6 tumor-bearing mice to determine whether CD4+ T cells contributed to the anti-tumor efficacy of M7824. Ninety-nine percent reduction of CD4+ T cells (Supplementary Figure 4A) led to tumor rejection in 100% of the M7824-treated mice (Supplementary Figure 4B-D). Further interrogation of this phenomenon revealed that EMT6 tumors, even in the absence of M7824 treatment, are highly susceptible to CD4 depletion, as 90% of PBS-treated mice underwent complete tumor rejection when CD4+ T cells were depleted (Supplementary Figure 4E-G). Similar observations have been reported in other tumor models.^30^

### M7824 induces a more active CD8+ and NK phenotype in EMT6 tumors

**Figure 5.**
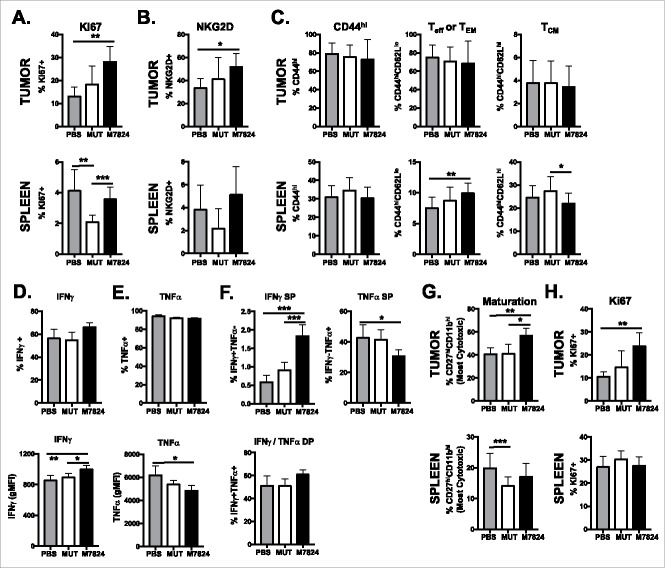
M7824 induces a more active CD8+ T and NK cell phenotype in the TME. EMT6 tumor-bearing mice were treated as in Figure 1. (A, B, C, G, H) Immune subsets in the tumor (top panels) or spleen (bottom panels) were examined 15 days after tumor implant by flow cytometry. CD8+ T cell expression of Ki67 (A), NKG2D (B), and maturation (CD44/CD62L) (C) were examined as well as NK cell maturation (CD27/CD11b) (G) and expression of Ki67 (H). Data combined from 2–3 independent experiments, n = 3-10 mice. (D, E, F) Isolated splenic CD8+ T cells were restimulated *ex vivo* with PMA and ionomycin for 4 hours. Expression of IFNγ (D, F) and TNFα (E, F) was determined by flow cytometry. Frequency of IFNγ single-producers (SP), TNFα SP, or IFNγ/TNFα double-producers (DP) in (F). Data are representative of 2 independent experiments, n = 5 mice. All graphs show mean ± SD.

As CD8+ T cells and NK cells were required for anti-tumor effects of M7824, we examined these cell types in the tumor and spleen of EMT6 tumor-bearing mice after treatment with M7824. There were no changes to frequency or number of CD8+ T cells in the tumor or spleen upon M7824 treatment (Supplementary Figure 5A). However, there was a greater frequency of Ki67+ CD44^hi^ CD8+ T cells in the tumor, indicating a greater proliferative capacity of these cells (Fig. 5A). In addition, there was a trend toward a larger population of CD44^hi^ CD8+ T cells that also expressed NKG2D in the TME (Fig. 5B), suggesting the accumulation of an activated, innate-like CD8+ T cell^31^^,^^32^ in the TME. These phenotypic effects were exclusive to M7824-treated tumors, as M7824mut did not induce these changes (Fig. 5A, B). In contrast to the tumor, there was a significant increase in the frequency of effector (T_eff_) or effector memory (T_EM_) CD8+ T cell populations in the spleens of M7824-treated mice (Fig. 5C). Frequency of central memory (T_CM_) CD8+ T cells was not increased with M7824 treatment (Fig. 5C). While we observed similar increases to the Ki67+ CD44^hi^ CD8+ T cell population in the spleen as in the tumor, there was no increase in the frequency of NKG2D+ cells in M7824-treated mice (Fig. 5A, B).

In order to examine the function of CD8+ T cells upon M7824 treatment, we restimulated isolated CD44^hi^ CD8+ T cells from the spleen with PMA and ionomycin. While the total frequency of IFNγ+ CD8+ T cells did not increase with treatment (Fig. 5D, upper panel), M7824-treated CD8+ T cells produced more IFNγ on a per cell basis (Fig. 5D, lower panel). In addition, a smaller frequency of CD8+ T cells from M7824-treated mice produced tumor necrosis factor alpha (TNFα) (Fig. 5E). This led to the overall frequency of IFNγ-single-producing CD8+ T cells to increase and TNFα-single-producing CD8+ T cells to decrease while having no effect on the IFNγ/TNFα double-producers (Fig. 5F). M7824mut treatment did not alter cytokine production of CD8+ T cells upon restimulation (Fig. 5D-F). These data suggest that M7824 treatment does increase the activity of CD8+ T cells, specifically skewing the population to produce IFNγ.

The effect of M7824 on NK cell phenotype was similar to that seen on CD8+ T cells. While there were no major changes in frequency or number of NK cells in the tumor or spleen (Supplementary Figure 5B), tumor-infiltrating NK cells displayed a more active, cytotoxic phenotype. M7824 treatment expanded the population of the most cytotoxic NK cell subset (CD27^hi^CD11b^hi^) (Fig. 5G). The frequency of Ki67+ NK cells also increased upon M7824 treatment (Fig. 5H). These effects were not observed in M7824mut-treated tumors or in the spleens of M7824-treated mice (Fig. 5G-I).

We did not observe significant changes to frequency or number of total CD4+ T cells (Supplementary Figure 6A), T_reg_ (Supplementary Figure 6B), or myeloid-derived suppressor cell (MDSC) populations (Supplementary Figure 6C-E) in the tumor with M7824 treatment. Together, these results suggest that M7824 treatment elicits its anti-tumor activity by increasing activation of CD8+ T cells and NK cells.

### M7824 treatment induces phenotypic changes to non-immune tumor-associated cells in the TME

EMT6 tumor cells produce high levels of TGFβ.^33^ Given TGFβ’s ability to work as both an autocrine and paracrine factor,^34^ sequestration of TGFβ in the TME by M7824 (Fig. 1), as well as cytokine production by tumor-infiltrating lymphocytes (TILs) (Fig. 5), could lead to phenotypic changes to non-immune cells in the TME, such as tumor and stromal cells. No changes to non-immune CD45 negative cells were observed within 1 week following M7824 treatment (data not shown). However, 1 week after the final M7824 treatment (day 21), we observed a 3-fold increase in the expression of PD-L1 and MHC class II in the TME CD45 negative population (Supplementary Figure 7A, B). No changes were observed in MHC class I expression as almost 100% of these cells expressed MHC class I in PBS-treated mice (Supplementary Figure 7C, D). Thus M7824 treatment does have an effect on tumor cell phenotype in the TME.

### M7824 complete responders display a distinct immune compartment as compared to non-cured mice

Seventeen to 40% of M7824-treated mice underwent complete EMT6 tumor rejection (Figs. 3,4), and this effect was apparent in the immune compartment 35 days after tumor implant. M7824-cured mice displayed a splenic immune compartment typical of mice that have undergone immune resolution. The total number of splenocytes (Supplementary Figure 8A), CD8+ T cells (Supplementary Figure 8B), and NK cells (Supplementary Figure 8D) were significantly decreased in M7824-complete responders. However, the frequencies of CD8+ T cells (Supplementary Figure 8B) and NK cells (Supplementary Figure 8D) were approximately double as compared to those in PBS- and M7824-tumor-bearing mice. In the CD8+ T cell compartment in complete responders compared to tumor-bearing mice, the frequency of naïve CD8+ T cells was significantly decreased by 30%, corresponding with an equally greater frequency of CD8+ T cells with an T_eff_ or T_EM_ phenotype (Supplementary Figure 8C). There were no differences in T_CM_ CD8+ T cell frequencies (Supplementary Figure 8C). In the NK cell compartment in cured mice, the frequency of the most cytotoxic NK cell population was decreased by 50%, while the most mature NK population increased as compared to tumor-bearing animals (Supplementary Figure 8E). NK cells from cured mice maintained a significant increase in the frequency of activated NKp46+ cells (Supplementary Figure 8F) as seen at earlier time points (Fig. 5). The differences observed between the CD8+ T and NK cells in cured versus non-cured mice suggest that a greater CD8+ T cell or NK cell response may be responsible for the ability to completely reject tumors.

### M7824 combination with Ad-TWIST vaccine improves anti-tumor response versus M7824 monotherapy

**Figure 6.**
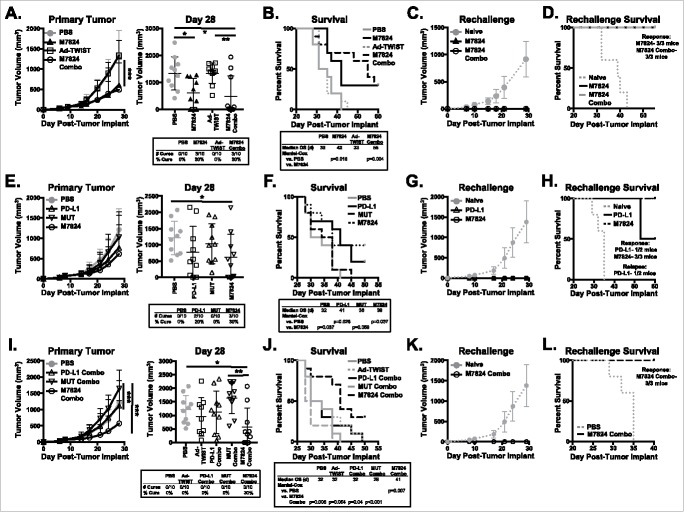
M7824 combination with Ad-TWIST improves anti-tumor efficacy versus M7824 or Ad-TWIST monotherapy. (A, B, E, F, I, J) EMT6 tumor-bearing mice were treated with PD-L1, MUT, or M7824 at days 10, 12, and 14 followed by vaccination with Ad-TWIST at days 16, 23, and 30. (A, E, I) Primary tumor growth curves (left panel) and tumor volumes of individual animals (right panel, inset: # of cured mice) show mean±SD. (B, F, J) Survival curves (inset: median OS in days) show % survival. (C, D, G, H, K, L) Cured mice from A, E, I, and 5 naïve Balb/c mice were implanted with EMT6 tumor cells at least 1 month after tumor rejection. (C, G, K) Tumor growth curves show mean±SD. (D, H, L) Survival curves (inset: # of mice with memory response or tumor relapse) show % survival. Data is representative of 2–3 independent experiments, n = 10 mice.

While tumor growth decreased and OS improved with M7824 treatment, only 17–40% of mice underwent complete tumor rejection (Figs. 3, 4). Since M7824 treatment enhanced the activation of CD8+ T cells, we hypothesized that combination therapy with a vaccine targeting a tumor-associated antigen (TAA) would improve anti-tumor efficacy. Thus, we combined M7824 with an adenovirus vaccine encoding the murine tumor-associated antigen TWIST1 (Ad-TWIST), which is expressed by EMT6 tumors (Supplementary Figure 9). TWIST1 is involved in EMT, metastasis, and angiogenesis,^35^^,^^36^ and previous studies have shown that vaccination against TWIST1 decreases murine tumor growth and spontaneous metastasis.^37^^,^^38^ EMT6 tumor-bearing mice were administered M7824 followed by three doses of Ad-TWIST weekly. While vaccination alone did not affect tumor growth or median OS as compared with PBS treatment, the combination of M7824 with Ad-TWIST significantly reduced the tumor burden (Fig. 6A) and increased median OS by 40% (Fig. 6B). While the decrease in tumor burden was similar between M7824 monotherapy and M7824/Ad-TWIST combination therapy (Fig. 6A), the combination of M7824 and Ad-TWIST did increase median OS by 31% compared to M7824 monotherapy (Fig. 6B). Both M7824 monotherapy and M7824/Ad-TWIST combination therapy induced the generation of protective memory, as all cured mice remained tumor-free after rechallenge (Fig. 6C, D). These results support the use of M7824 with cancer vaccines to improve anti-tumor efficacy.

### Dual targeting of TGFβ and PD-L1 is required for synergistic activity of M7824 in combination with Ad-TWIST vaccine

The αPD-L1 moiety of M7824 is based on avelumab. Treatment of EMT6 tumor-bearing mice with αPD-L1/avelumab led to a reduction in tumor burden (Fig. 6E) and increases in median OS (Fig. 6F) similar to that observed with M7824 monotherapy. M7824 promoted a robust generation of protective memory, as 3/3 M7824-cured mice were protected against EMT6 tumor rechallenge (Fig. 6G, H). Only 1/2 αPD-L1-cured mice were protected from tumor rechallenge (Fig. 6G, H). The anti-tumor efficacy of both αPD-L1 and M7824 outperformed M7824mut (Fig. 6E, F).

When αPD-L1 was given in combination with Ad-TWIST vaccine, however, the anti-tumor efficacy of αPD-L1 therapy was lost. αPD-L1/Ad-TWIST combination-treated mice had tumor burden (Fig. 6I) and median OS (Fig. 6J) similar to that found in PBS- and M7824mut-treated animals. This is in distinct contrast to the increased OS observed in M7824/Ad-TWIST combination-treated mice versus PBS- and M7824mut-treated mice (Fig. 6J). Additionally, the combination of M7824mut with vaccine did not increase anti-tumor efficacy relative to PBS treatment (Fig. 6I, J). These results demonstrate that M7824, but not αPD-L1, is amenable to combination with cancer vaccines. Importantly, these results demonstrate that both blockade of PD-L1 and TGFβ sequestration are required for synergistic anti-tumor efficacy in combination with a cancer vaccine.

## Discussion

Activating the immune system for therapeutic benefit against cancer has recently become a reality with the use of immune checkpoint-targeted mAbs, such as αPD-L1/αPD-1 antibodies. However, many patients see no clinical benefit with αPD-L1/αPD-1 therapy, even if their tumors express the targeted ligand.^1^ One explanation for this is the presence of other molecules in the tumor microenvironment, such as the immunosuppressive cytokine TGFβ. In these studies, we show that dual inhibition of PD-L1- and TGFβ-mediated suppressive pathways by the novel agent M7824 produced substantial anti-tumor efficacy against murine models of breast and colon carcinoma. M7824 decreased tumor burden and increased OS versus control-treated mice. Importantly, both the ability to block PD-L1 and sequester TGFβ was required for the efficacy of M7824, as TGFβ sequestration alone by M7824mut did not improve tumor responses. This is in contrast to other studies showing that blocking TGFβ1 and TGFβ2 alone was sufficient to improve anti-tumor efficacy in murine tumor models.^39^ However, those studies began treatment on the day of tumor implant, which may improve immune cell priming before tumor establishment and lead to altered development of an immunosuppressive microenvironment.

Blocking PD-L1 played a major role in M7824-mediated therapy, as treatment with the parent molecule avelumab (αPD-L1), which only blocks PD-L1, showed similar anti-tumor efficacy. However, M7824 combination with Ad-TWIST vaccine further improved OS versus M7824 monotherapy, similar to results in studies using a TGFβ blocking antibody in combination with an αPD-1 antibody and vaccine.^39^ This synergistic effect was not observed with αPD-L1 and vaccine combination therapy. In fact, the combination of αPD-L1 and vaccine completely abrogated the anti-tumor efficacy of αPD-L1. How M7824 is able to synergize with a therapeutic cancer vaccine while αPD-L1 therapy does not is currently under investigation. Preliminary data suggest that the distribution of CD8+ T cells to the spleen and lymph nodes may be decreased on αPD-L1 but not M7824 treatment (data not shown). This may prevent proper CD8+ T cell priming upon vaccination after αPD-L1 treatment. Alternatively, the presence of TGFβ in both the periphery as well as the tumor of αPD-L1-treated animals may prevent CD8+ T cell activation upon vaccination, which M7824 can overcome as it both sequesters plasma TGFβ1 and prevents TGFβ-induced signaling. TGFβ is also known to regulate the development and function of T_reg_,^28^ which play an important role in CD8+ T cell activation and function. Thus, it is possible that M7824 may decrease T_reg_ function during CD8+ T cell activation and priming after vaccination. Previous studies have shown that M7824 treatment of human T_reg_ decreases their suppressive function.^40^ Collectively, the results presented here suggest that the use of the novel bifunctional agent M7824 does have potential advantages versus pre-existing αPD-L1/αPD-1 therapies and should be further investigated for use in the clinic, including in combination with therapeutic cancer vaccines as well as other immunotherapies.

M7824 combines the ability to block PD-L1 and sequester TGFβ into a single agent. Whether M7824, as a single bifunctional molecule, elicits better anti-tumor responses than the use of αPD-L1 in combination with a TGFβ inhibitor or TGFβ-targeted mAb remains to be explored. However, we hypothesize that M7824 will have increased anti-tumor efficacy since we have shown that it accumulates in the tumor by binding to PD-L1. By increasing the concentration of M7824 at the tumor site, it allows for local sequestration of TGFβ, which is produced in high levels by both tumor and tumor-associated immune cells.^13^^,^^41^ Importantly, our results show that even though M7824 significantly decreases plasma TGFβ1 levels, it still has the ability to suppress TGFβ-dependent signaling in the TME. This was dependent on accumulation of M7824 in the tumor, as M7824mut decreased TGFβ1 levels in the plasma but did not bind to TME-expressed PD-L1 and did not decrease TGFβ-dependent signaling in the TME. Why M7824 treatment decreased TGFβ-dependent activation of SMAD2 but not SMAD3 remains to be determined. However, it is known that SMAD2 and SMAD3 have different thresholds and kinetics of activation upon TGFβ stimulation.^42-44^ Thus, it possible that SMAD3 is also affected by M7824, as has been seen in human tumor cell lines.^45^ Targeting TGFβ sequestration to the tumor site may also reduce the potential adverse effects of using a systemic TGFβ inhibitor, such as cardiac toxicity, development of neoplasms, and re-activation of dormant cancer cells seen in preclinical models with some TGFβR-targeted small molecule inhibitors.^23^

TGFβ is a known suppressor of tumor progression early in tumor development.^13^^,^^14^ Some studies have shown that lower TGFβ serum level correlates with worse prognosis,^46^ and loss of TGFβ signaling accelerates tumor growth. In our studies, sequestration of TGFβ did not promote tumor growth.^20^^,^^22^^,^^47^ In fact, M7824 displayed potent anti-tumor activity in multiple murine tumor models. This could be due to the treatment schedule of M7824, which was administered after the tumors were well-established. In addition, the administration of M7824 to EMT6, MC38, or 4T1 tumor cells *in vitro* induced no changes to tumor cell proliferation over the course of 5 days (data not shown).

In our studies, the anti-tumor effects of M7824 were highly dependent on CD8+ T cell and NK cells. Loss of these cell populations by antibody-mediated depletion abrogated the therapeutic capacity of M7824. Immunophenotypic analysis supported this finding, as M7824 promoted an activated CD8+ T and NK cell phenotype in both tumor- and non-tumor-bearing mice. This is in agreement with the effects of blocking PD-L1/PD-1 and TGFβ immunosuppressive pathways during T and NK cell responses.^48-50^ Although the PD-L1/PD-1 interaction and TGFβ are known to regulate T and NK cell exhaustion and anergy,^48^^,^^51^^,^^52^ no decreases were observed in the expression of phenotypic markers associated with exhaustion, such as PD-1, Tim-3, or VISTA (data not shown). As functional immune exhaustion is dependent on a balance of activation and inhibitory/exhaustive signals,^53^ it is possible that the immune-activating capabilities of M7824 are able to overcome any existing expression of these receptors. Alternatively, since these “exhaustion”-related receptors are normally expressed upon immune cell activation to prevent excessive immune responses,^53^ a distinct reduction in their expression may not be observed due to the immune-activating nature of M7824.

The expression of TGFβ receptor on naïve CD8+ T cells maintains a stimulation threshold restricting T cell activation. Genetic loss of TGFβ receptor or suppression of TGFβ signaling decreases this threshold and allows for activation of CD8+ T cells against lower affinity antigens,^54^ such as TAAs. Thus, sequestration of TGFβ by M7824 may increase the diversity of responding CD8+ T cell clones during anti-tumor immune responses. This is important, as a more diverse T cell repertoire promotes successful and long-lasting anti-tumor immunity.^55-57^ Studies investigating T cell repertoire diversity during M7824 treatment may also provide insight into rational immunotherapy combination design. For example, we observed improved anti-tumor responses when M7824 was given in combination with a cancer vaccine. Vaccination against TAAs in cancer patients is known to induce greater tumor antigen-specific immune responses and can also lead to responses against other TAAs not encoded by the vaccine.^58^^,^^59^ By lowering the T cell activation threshold with TGFβ sequestration and supporting expanded T cell activation and function by blocking PD-L1, M7824 may further improve these antigen-specific responses and lead to better clinical outcome.

In conclusion, the studies presented here demonstrate that dual targeting of PD-1/PD-L1 and TGFβ immunosuppressive pathways with the novel bifunctional agent M7824 promotes potent anti-tumor responses in murine models of human solid carcinomas. Furthermore, these results support clinical development of M7824 as both a monotherapy and in combination with other immunotherapies, such as therapeutic cancer vaccines. A phase I clinical study of M7824 in heavily pretreated patients with advanced solid carcinomas is ongoing (NCT02517298).^29^ M7824 was shown to be well-tolerated, with adverse events similar to those seen with other αPD-L1/PD-1 mAbs. In addition, there was evidence of clinical efficacy seen across all dose levels (0.3-20 mg/kg). This included one ongoing confirmed complete response in a cervical cancer patient (10 mg/kg), one durable partial response in a patient with pancreatic cancer (3 mg/kg), and two cases of prolonged stable disease in pancreatic (3 mg/kg) and carcinoid (1 mg/kg) cancer patients.^29^ Phase II studies utilizing M7824, including a study combining M7824 and a therapeutic cancer vaccine targeting prostate specific antigen (PSA) or carcinoembryonic antigen (CEA) plus mucin 1 (MUC1), are currently planned or ongoing in multiple tumor types.

## Materials and methods

### Tumor cell lines

Murine breast carcinoma cells EMT6 and 4T1 were obtained from American Type Culture Collection (ATCC) and 4T1-pSMAD2-luc was kindly provided by EMD Serono. All cell lines were cultured according to the manufacturer's instructions. Murine colon carcinoma MC38 cells are as described.^60^ All cell lines were free of *mycoplasma* as determined by MycoAlert Mycoplasma Detection Kit (Lonza) and were used at low passage numbers.

### Reagents

The following biological agents were generously provided by EMD Serono under a Cooperative Research and Development Agreement with NCI: avelumab (designated as αPD-L1), a fully human αPD-L1 (IgG1); M7824, a fully human bifunctional molecule comprised of the extracellular domain of human TGFβRII (TGFβ Trap) linked to the C-terminus of αPD-L1 heavy chain; and M7824mut, a fully human molecule comprised of human TGFβRII bound to a mutated αPD-L1 moiety. Recombinant murine TGFβ1 was from R&D Systems and recombinant murine IFNγ was obtained from PeproTech. CD8 (2.43) and CD4 (GK1.5) depletion antibodies were obtained from BioXcell and the NK (anti-asialo-GM1) depletion antibody was from Wako Chemicals. Adenovirus encoding the murine TWIST1 gene (Ad-CMV-TWIST, referred to as Ad-TWIST) was from Vector Biolabs.

### Flow cytometry and antibodies

Preparation of cells for flow cytometry was performed using the BD Cytofix/Cytoperm Kit (BD Biosciences) according to the manufacturer's instructions. Anti-H-2D^d^ (34-2-12), -H-2K^d^ (SF1-1.1), -I^A^/I^E^ (M5/114), -GR1 (RB6-8C5), -FoxP3 (R16-715), -CD62L (MEL-14), -CD44 (IM7), -CD3Ɛ (2C11), -CD274 (MIH5), -CD19 (1D3), -CD11b (M1/70), -IFNγ (XMG1.2), -TNFα (MPG-XT22) were purchased from BD Biosciences. Anti-CD8α (53-6.7), -Ki67 (SolA15), -Eomes (Dan11Mag), -FoxP3 (FJK-16s), -CD69 (H1.2F3), -NKp46 (29A1.4), -NKG2D (CX5), -CD279 (J43) were from eBioscience. Anti-CD8β (53-5.8), -VISTA (MH5A), -Tim-3 (B8.2C12), -Ly6C (HK1.4), -Ly6G (1A8), -CD49b (DX5), -CD45.2 (104), -CD4 (RM4-5, RM4-4), -CD25 (PC61), -CD122 (TMβ1) were purchased from Biolegend. Anti-SMAD2/3 (D7G7) and anti–phospho-SMAD2/3 (D27F4) were from Cell Signaling Technology, and anti-TGFβRII was from R&D Systems. Anti-human (H+L) secondary antibody and Live/Dead Fixable Dead Cell Stain were from Invitrogen. Matched isotypes were obtained from the aforementioned manufacturers. Flow cytometry (≥ 1 × 10^5^ events acquired per sample) was performed on a BD FACSVerse or LSRII Fortessa flow cytometer (Beckton Dickinson) and analyzed with FlowJo FACS Analysis Software (Treestar). Cell populations were identified as follows: CD8+ T cells: live/CD45.2+/CD3+/CD8+; CD4+ T cells: live/CD45.2+/CD3+/CD4+/FoxP3 negative; CD4+ T_reg_: live/CD45.2+/CD3+/CD4+/FoxP3+; NK cells: live/CD45.2+/CD3-/CD49b+; Gr1+ cells: live/CD45.2+/CD11b+/Gr1+; Granylocytic MDSC/Neutrophils: live/CD45.2+/CD11b+/Ly6Ghi/Ly6Clo; Monocytic MDSC/Monocytes: live/CD45.2+/CD11b+/Ly6Glo/Ly6Chi; non-immune, tumor cell compartment: live/CD45 negative. All frequencies of cells expressing phenotypic proteins were generated by subtracting the frequency of respective isotype, typically set between 1–5%.

### Mice

Six- to 10-week old female Balb/c and C57BL/6 mice were obtained from the National Cancer Institute's Frederick Cancer Research Facility, Frederick, MD, and were maintained in microisolator cages under specific pathogen-free conditions in accordance with the Association for Assessment and Accreditation of Laboratory Animal Care (AAALAC) guidelines. All experimental studies were carried out under approval of the NIH Intramural Animal Care and Use Committee.

### Murine tumor studies

For murine breast carcinoma studies, 2.5 × 10^5^ EMT6 tumor cells or 5 × 10^4^ 4T1 tumor cells were orthotopically implanted subcutaneously (s.c.) into the mammary fat pad of female Balb/c mice. For murine colon carcinoma studies, 5 × 10^5^ MC38 tumor cells were implanted s.c. into the right flank of female C57BL/6 mice. Mice were randomized based on tumor size and treatments were initiated when tumors reached 50–100mm^3^. Mice received three doses of 400μg αPD-L1, 492μg M7824mut, or 492μg M7824 (all equivalent to 20 mg/kg) via intraperitoneal injection (i.p.) in all experiments. For vaccine combination studies, animals received 10^10^ Ad-TWIST virus particles (VP) s.c. beginning 7 days after the initiation of treatment with αPD-L1, M7824mut, or M7824 and then weekly for a total of three doses. All tumors were measured twice weekly using calipers and tumor volumes were determined using the formula (length^2^ × width)/2. For tumor rechallenges, EMT6 or MC38 tumor cells were implanted at the numbers and locations listed above into cured mice (and naïve controls) at least 1 month after tumor rejection.

### 4T1 lung metastasis

For quantitative analysis of 4T1 lung metastasis in tumor-bearing mice, lungs were processed and plated as previously described, based on 4T1 exclusive resistance to 6-thioguanine (6-TG).^37^^,^^61^ Briefly, lung single-cell suspensions were obtained through enzymatic digestion and plated in 6-TG containing medium at 37 °C/5%CO_2_. After 10–14 days in culture, cell colonies resulting from 4T1 single-cell clonal expansion were fixed with methanol, washed, stained with methylene blue, and enumerated.

### Depletion studies

100μg anti-CD4 (GK1.5) or anti-CD8 (2.43) depletion antibodies were administered i.p. to tumor-bearing mice on days 5, 6, and 7 post-tumor implant and then once weekly for the duration of the experiment. 25μl anti-NK (anti-asialo-GM1) depletion antibody was administered in a total of 100μl PBS i.p. on days 5 and 7 post-tumor implant followed by every 3 days until day 18 and then every 5 days through the duration of the experiment. Anti-CD8 and anti-NK antibodies were given in combination according to the aforementioned schedule. Blood was obtained weekly from three mice per group to determine immune cell population depletion efficiency by flow cytometry.

### Flow cytometric analysis of immune and tumor cells

Spleens and lymph nodes (cervical, brachial, axillary and inguinal) were harvested, smashed using 120μM nylon sheets, filtered through a 70μM filter, and subjected to ACK lysis. Tumors were harvested, cut into 2 mm pieces and processed using the gentleMACS Dissociator according to the manufacturer's instructions (Miltenyi Biotec). After digestion, single cell suspensions were filtered through a 70μM filter. TILs were enriched using a 44%/67% Percoll (GE Healthcare) gradient. All cell counts were performed using 123count eBeads (eBioscience). Approximately 1–10 × 10^6^ immune cells were stained for flow cytometry.

### CD8+ T cell restimulation

CD8+ T cells were isolated by negative selection from spleens of EMT6-tumor bearing mice at day 15 post-tumor implant using the CD8α+ T Cell Isolation Kit (Miltenyi Biotec) according to the manufacturer's instructions. 1 × 10^6^ CD8+ T cells were stimulated with nothing or 50 ng/ml PMA and 500 ng/ml ionomycin for 4 hours. Intracellular IFNγ and TNFα production were detected by flow cytometry.

### Immunoblot analysis

Protein lysates from MC38, EMT6 or 4T1 tumor cell lines were prepared with Cell Lysis Buffer (Cell Signaling) supplemented with 1 mM phenylmethanesulfonyl fluoride (Sigma-Aldrich). Lysates from 5 × 10^5^ cells were resolved by SDS-PAGE and transferred to PVDF membranes using standard techniques and blocked using 5% bovine serum albumin (BSA). Membranes were incubated overnight at 4 °C with either anti-TWIST (Twist2C1a) from Santa Cruz Biotechnology or β-actin from Cell Signaling. Membranes were then incubated with appropriate secondary antibodies conjugated with IRDye-680 and visualized using the Odyssey Infrared Imaging System (LI-COR Biosciences).

### Quantitation of TGFβ isoforms in mouse plasma

Platelet-poor plasma was collected as previously described.^62^^,^^63^ TGFβ isoforms were measured using TGFβ Premixed Magnetic Luminex Performance Assay kit (R&D Systems, Cat# FCSTM17) according to the manufacturer's instructions. Quantification and analysis were performed with the Bio-Plex MAGPIX reader and Bio-Plex Manager software (Bio-Rad). Detection of TGFβ isoforms was performed both with and without acid-activation to determine the total level of active TGFβ1/2/3 (without acid activation) and total TGFβ1/2/3 (with acid activation). The limit of detection for TGFβ1 was 35.187 pg/ml, TGFβ2 was 16.953 pg/ml, and TGFβ3 was 55.574 pg/ml.

### Measurement of SMAD activation in mouse tumor samples

EMT6 tumors were sectioned, weighed, and snap-frozen in liquid nitrogen prior to analysis of SMAD2/3/1/5 activation by capillary Western blot. Analyses were performed using the reagents provided by the vendor as previously described.^64^ Briefly, tumor lysates (approximately 40 ng protein) were mixed with 1x SDS master mix containing sample buffer, DTT and fluorescently labeled standards and were heated at 70 ˚C for 10 minutes before being loaded into Peggy Sue (ProteinSimple) for analysis. Chemiluminescence was captured and quantified. Digital images were analyzed by Compass software (ProteinSimple). Target protein quantities were determined based on peak areas. The antibodies used in the study were: anti-Smad1/5 pS463/465, anti-Smad5, anti-Smad2 & anti-Smad3 (Cell Signaling Technology); anti-Smad3 pS423/425 & anti-Smad1 (Abcam), anti-Smad2 pS465/467 (Millipore), loading control anti-Vinculin (Sigma Aldrich), and horseradish peroxidase (HRP) conjugated anti-rabbit or anti-mouse secondary antibodies (ProteinSimple or Jackson ImmunoResearch).

### Luciferase assay

Detection of luciferase was performed using Bright-Glo Luciferase Assay System (Promega) according to the manufacturer's instructions. In experiments where cells were pre-treated with biologics prior to TGFβ addition, luciferase was quantified at 1 hour post TGFβ addition, corresponding to the approximate peak of SMAD2 phosphorylation (Supplemental Figure 1C). Alternatively, in experiments where cells were treated with biologics after TGFβ addition, luciferase was quantified at 6 hours after TGFβ addition to allow for significant degradation of luciferase protein (t_1/2_ = 2 hours) following cessation of TGFβ-induced SMAD2 phosphorylation.

### Statistics

Statistical analyses were performed in GraphPad Prism 7 (GraphPad Software). Unless otherwise stated, one-way ANOVA with Tukey's multiple comparisons was used for statistical analysis for data presented in bar graphs or scatter plots. Two-way ordinary ANOVA was used to analyze tumor growth curves. Survival was analyzed using Log-rank (Mantel-Cox) test. Statistical significance was set at *p* <0 .05. *p < 0.05, **p < 0.005, ***p < 0.001.

## Supplementary Material

Supplemental Material
