# Supplementary material for: M7824, a novel bifunctional anti-PD-L1/TGFβ Trap fusion protein, promotes anti-tumor efficacy as monotherapy and in combination with vaccine
Source: Oncoimmunology. 2018 Feb 14;7(5):e1426519. doi: 10.1080/2162402X.2018.1426519 (PMC5927523; doi:10.1080/2162402X.2018.1426519)
Supplement: Supplemental Material [file koni-07-05-1426519-s001.zip › 2._Knudson_Erratum_Figure_S1.pdf]

Figure S1

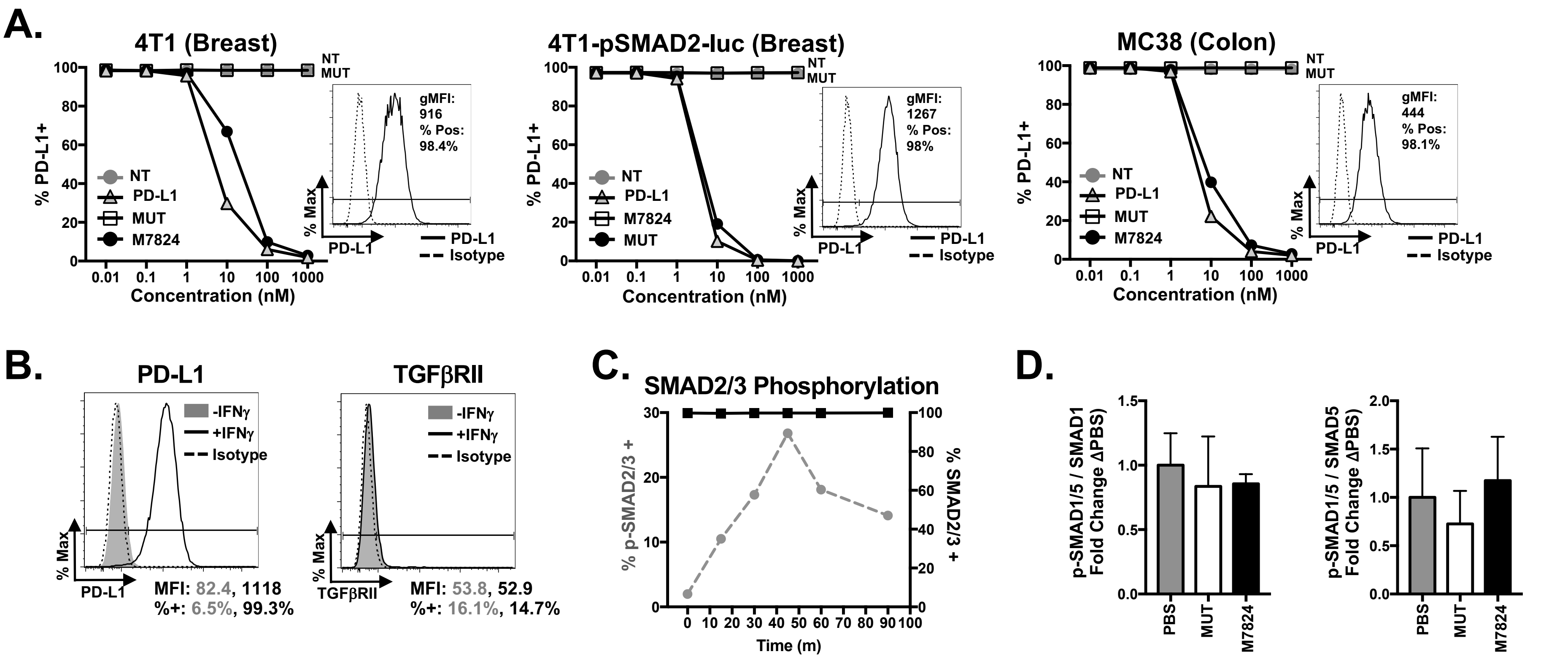

Figure S1. (A) M7824 binds cell surface PD-L1 on murine tumor cell lines *in vitro.* 4T1, 4T1-pSMAD2-luc, and MC38 tumor cells were exposed to 100nM IFNγ for 24 hours followed by treatment with nothing (no treatment-NT), anti-PD-L1 (PD-L1), M7824mut (MUT), or M7824 for 30 minutes prior to analysis of surface PD-L1 expression by flow cytometry. Data represent 3 independent experiments.

(B,C) 4T1-pSMAD-luc cells express PD-L1 and TGFβRII and activate TGFβ signaling pathways upon TGFβ1 stimulation. 4T1-pSMAD2-luc tumor cells were left untreated or treated with100nM IFNγ for 24 hours. **(B)** PD-L1 and TGFβRII expression were determined by flow cytometry. **(C)** 4T1-pSMAD2-luc tumor cells were exposed to 2.5ng/ml TGFβ1 and level of total and phosphorylated SMAD2/3 was determined by flow cytometry. Data represent 3 independent experiments.

(D) M7824 does not affect intratumoral SMAD1 or SMAD5 activation. EMT6 tumor cells were implanted as in Figure 1. When tumor volumes reached 500mm^3, mice were treated at days 17, 19, and 21 with MUT or M7824 i.p. 6 hours after the last treatment, phosphorylation and total level of SMAD1 and SMAD5 were determined by capillary Western blot. Data combined from 2 independent experiments, n=2-5 mice per experiment.
