# Supplementary material for: M7824, a novel bifunctional anti-PD-L1/TGFβ Trap fusion protein, promotes anti-tumor efficacy as monotherapy and in combination with vaccine
Source: Oncoimmunology. 2018 Feb 14;7(5):e1426519. doi: 10.1080/2162402X.2018.1426519 (PMC5927523; doi:10.1080/2162402X.2018.1426519)
Supplement: Supplemental Material [file koni-07-05-1426519-s001.zip › Pages from KNUDSON_Supplementary_Figures.pdf]

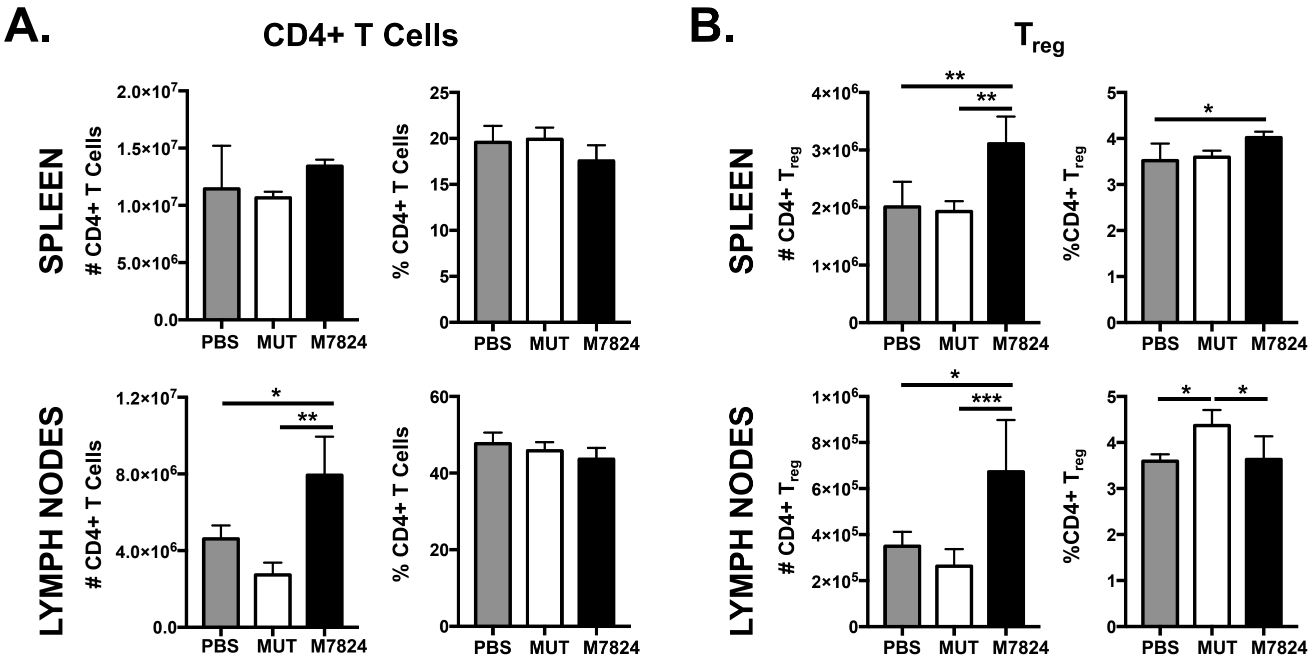

Figure S2. M7824 increases CD4+ T cell and Treg numbers in non-tumor-bearing mice. Naïve Balb/c mice received 3 doses of MUT or M7824 on day 0, 2, and 4. Immune populations in the spleen and lymph nodes 3 days after the last treatment were analyzed by flow cytometry. Graphs show frequency (of total live cells) and number of CD4+ T cells **(A)** and CD4+ T_{reg} **(B)** 3 days post-treatment. All graphs show mean$\pm$SD. Data combined from 2 independent experiments, n=3-5 mice per experiment.

A. B.

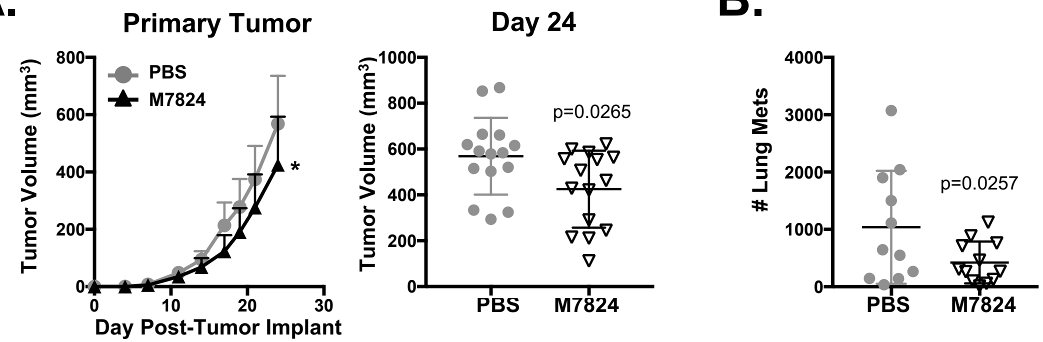

C.

Number of Mets in Lung	PBS (n=11)	M7824 (n=12)
≥ 2000	2/11 (18.2%)	0
≤ 1000	6/11 (54.5%)	11/12 (91.7%)
≤ 500	4/11 (36.4%)	8/12 (66.7%)
≤ 300	4/11 (36.4%)	6/12 (50%)
≤ 100	1/11 (9.1%)	3/12 (33.3%)
≤ 50	1/11 (9.1%)	1/12 (8.3%)
≤ 10	0	0
0	0	0
Median	645	286
Mean	1037	422

Figure S3. M7824 decreases 4T1 breast tumor cell metastasis. $5x10^4$ 4T1 tumor cells were orthotopically implanted into female Balb/c mice. Mice received 2 doses of 492μg M7824 i.p. at days 7 and 9 post-tumor implant. Twenty-four days after tumor implant, lungs were harvested and single-cell suspensions were plated with 6-TG for 14 days to visualize lung metastases. **(A)** Primary tumor growth curves (left panel) and tumor volumes of individual mice at day 24 (right panel) show mean±SD. Number of lung metastases in individual mice (mean±SD) shown in **(B).** Table showing the distribution of number of lung metastasis in **(C).** Data from 1 independent experiment, n=11-14 mice. Statistics in A (right panel) and B determined by a two-tailed t test.

Figure S4

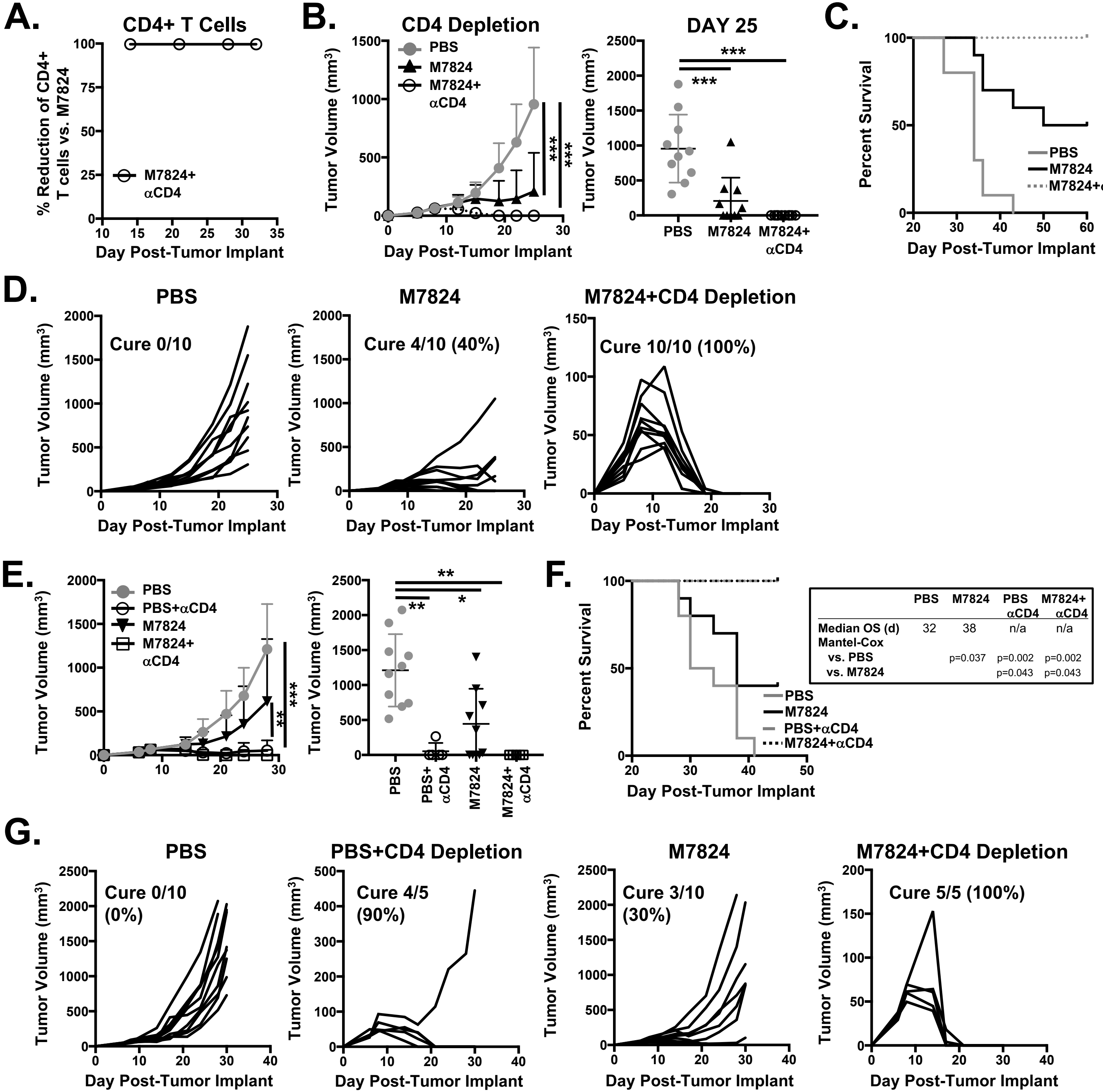

Figure S4. Depletion of CD4+ T cells leads to complete rejection of EMT6 breast tumors regardless of M7824 treatment. (A-D) EMT6 tumor-bearing mice were treated as in Figure 1 with PBS or M7824. M7824-treated mice underwent depletion of CD4 cells. **(A)** Depletion efficiency was determined in the blood weekly by flow cytometry. Graph (mean±SD) shows % reduction of CD4+ T cells versus M7824-treated mice (set to 0%). **(B)** Primary tumor growth curves (left panel) and tumor volumes of individual animals (right panel) show mean±SD. **(C)** Survival curves (inset shows median OS in days) show % survival. Growth of tumors in independent mice (inset shows # of cured mice) is shown in **(D).** Data represent 1 independent experiment, n=10 mice. **(E-G)** The study in (A) was repeated including CD4 depletion of PBS-treated mice. **(E)** Primary tumor growth curves (left panel) and tumor volumes of individual animals (right panel) show mean±SD. **(F)** Survival curves (inset shows median overall survival in days) show % survival. Growth of tumors in independent mice (inset shows # of cured mice) is shown in **(G).** Data represent 1 independent experiment, n=5-10 mice.

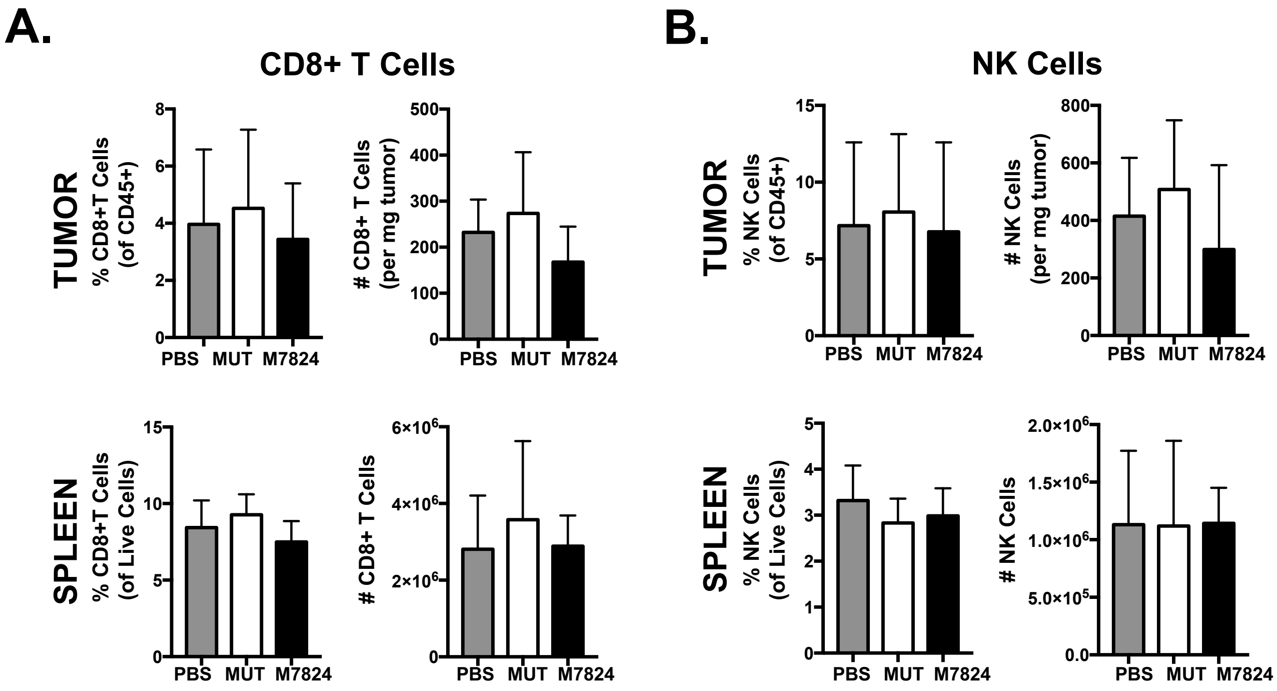

Figure S5. M7824 does not alter frequency or number of CD8+ T cell and NK cells in the tumor or spleen
Mice were treated as in Figure 5. Immune subsets in the tumor were examined 15 days after tumor implant. Frequency (of total CD45+ cells in the tumor and of total live cells in the spleen) and number of CD8+ T cells **(A)** and NK cells in the tumor and spleen were determined by flow cytometry. Graphs show mean±SD Data represent 2 independent experiments, n=5-10 mice per experiment.

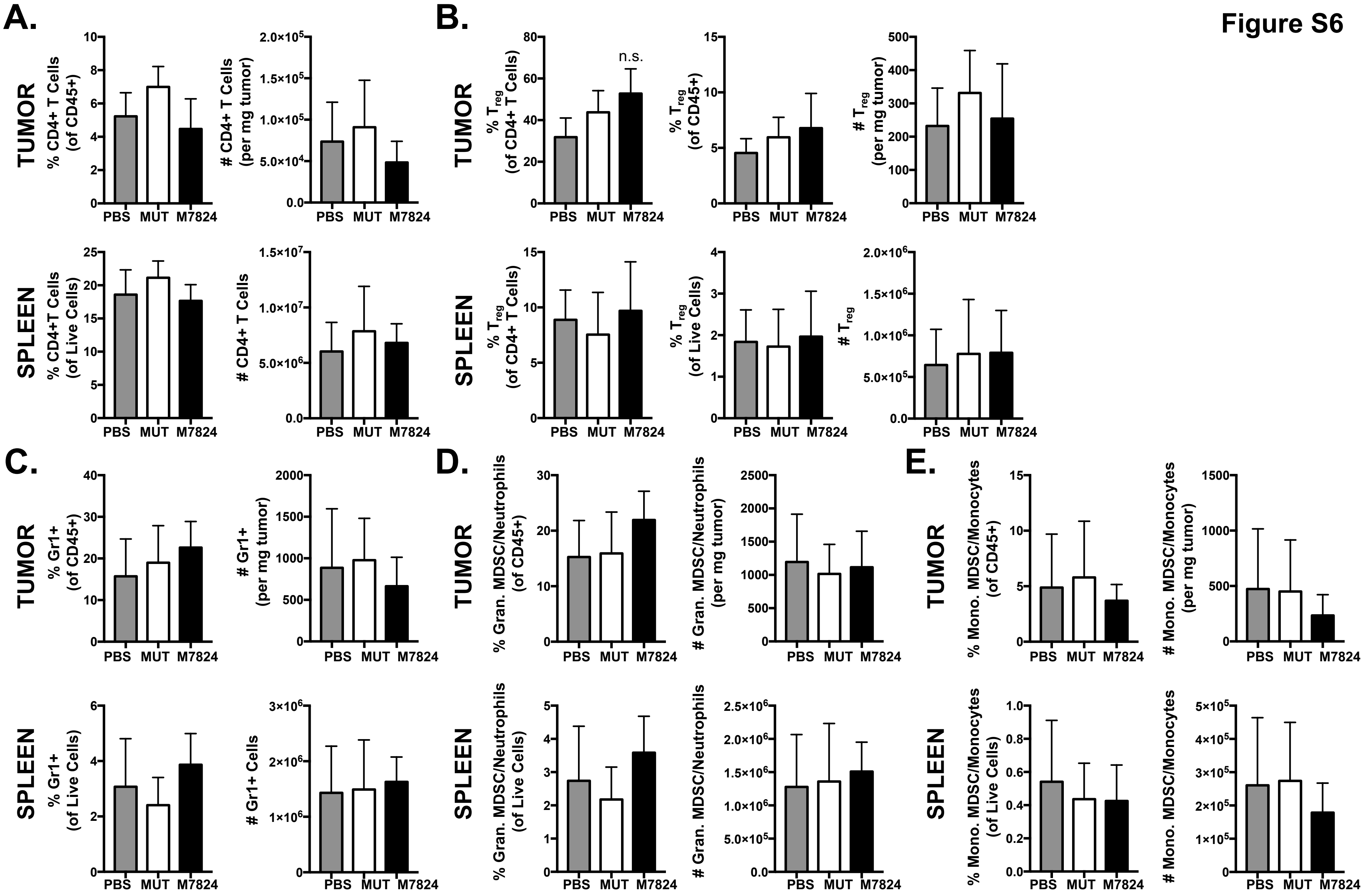

Figure S6. Effect of M7824 on CD4+ T cells and MDSC. Mice were treated as in Figure 5. Immune subsets in the tumor or spleen were examined 15 days after tumor implant. Frequency (of total CD45+ cells in the tumor and of total live cells in the spleen) and number of CD4+ T cells **(A)**, CD4+ T_{reg} **(B)**, Gr1+ MDSC **(C)**, granulocytic MDSC/neutrophils **(D)**, and monocytic MDSC/monocytes **(E)** in the tumor and spleen were determined by flow cytometry. Graphs show mean±SD. Data combined from 2 independent experiment, n=5-10 mice per experiment.

Figure S7

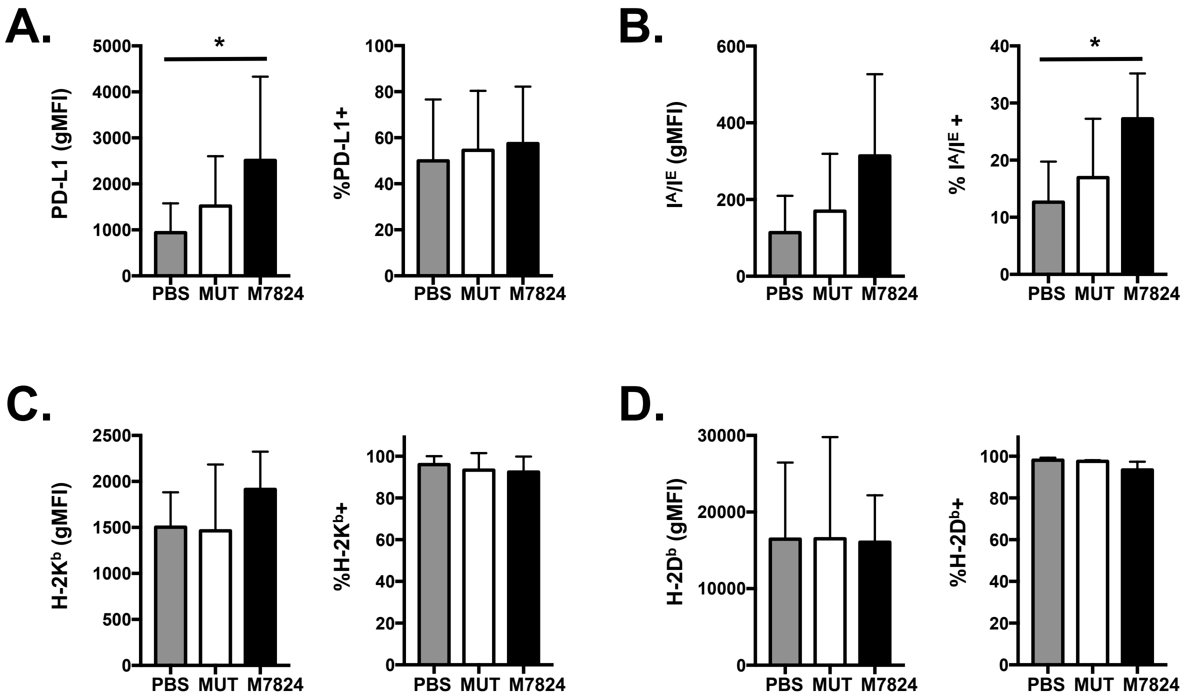

Figure S7. M7824 induces upregulation of MHCII and PD-L1 on non-immune tumor-associated cells. Mice were treated as in Figure 3. Twenty-one days after tumor implant, expression of surface PD-L1 **(A)**, MHC class II **(B)**, and MHC class I **(C,D)** on the primary tumor (CD45- cells) was determined by flow cytometry. All graphs show mean±SD. Data combined from 2 independent experiments, n=3-5 mice per experiment.

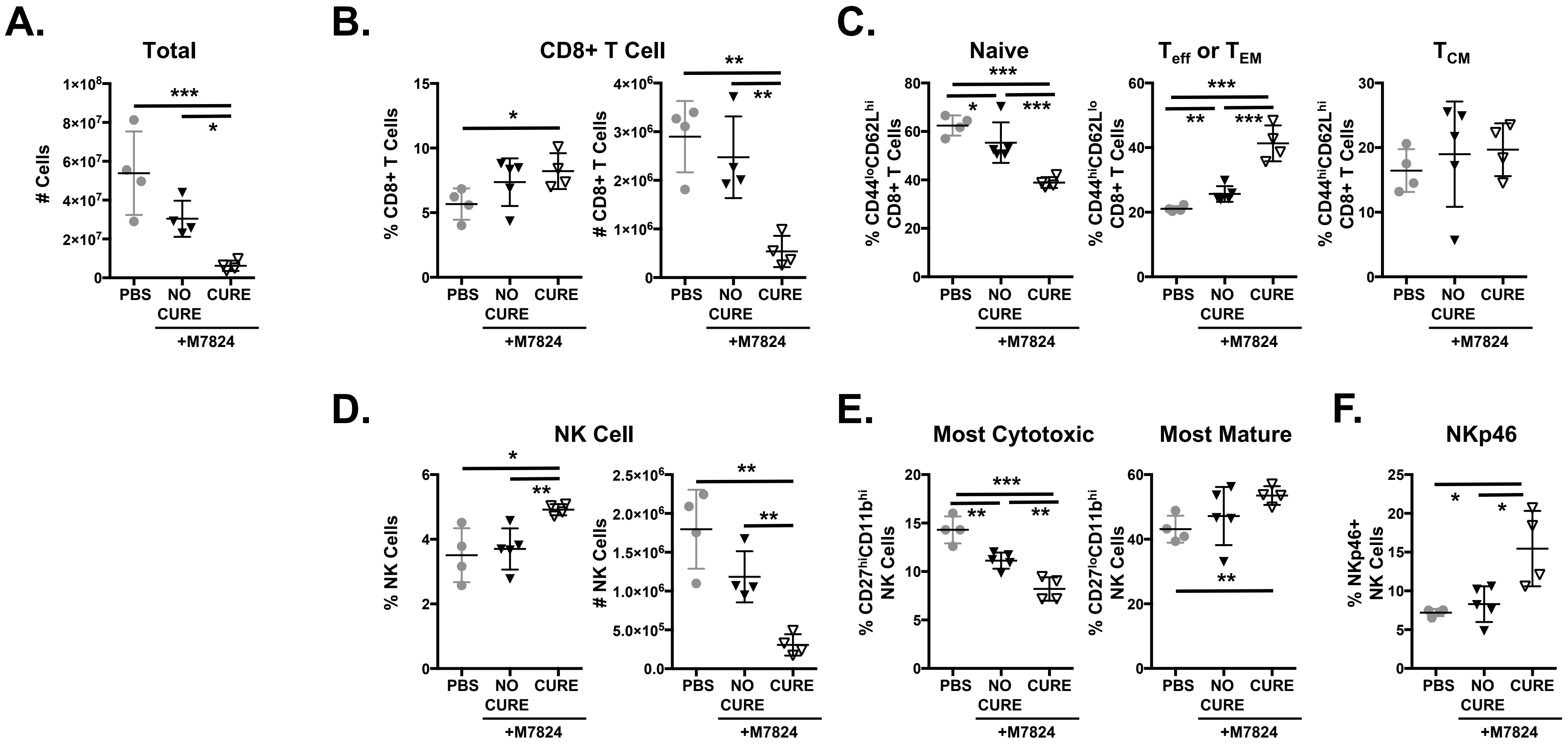

Figure S8. Phenotype of splenic immune subsets in cured vs. non-cured mice with M7824 treatment. Mice were implanted with EMT6 tumors as in Figure 3 and treated at days 9, 11, and 14 with PBS or M7824. Immune subsets in the spleen were examined 35 days after tumor implant in mice that did not (NO CURE) or did (CURE) undergo tumor rejection with M7824 treatment. Graphs show total cell numbers **(A)**, frequency (of total live cells) and number of CD8+ T cells **(B)** or NK cells **(D)** and phenotype of CD8+ T cells **(C)** or NK cells **(E,F)** in individual mice (mean±SD) as determined by flow cytometry. Data represent 1 independent experiment, n=4-5 mice.

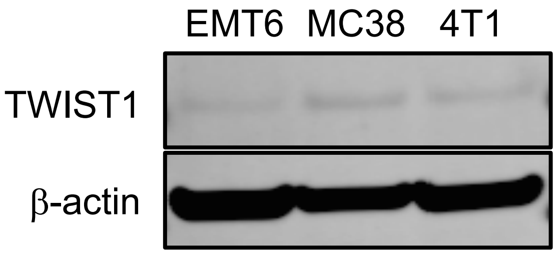

Figure S9. EMT6 tumor cells express TWIST. Immunoblot analysis of TWIST1 and β-actin in EMT6, MC38, and 4T1 murine tumor cells. Data from 1 experiment.
